# Stress as a mediator between abusive supervision and clinical nurses’ work outcomes

**DOI:** 10.1111/inr.12961

**Published:** 2024-03-18

**Authors:** Leodoro J. Labrague

**Affiliations:** ^1^ Clinical Assistant Professor Marcella Niehoff School of Nursing Loyola University Chicago Chicago Illinois USA

**Keywords:** Absenteeism, abusive supervision, job engagement, nursing, stress, turnover intention

## Abstract

**Aims:**

This study aimed to test whether stress could mediate the association between abusive supervision and nurses’ work engagement, absenteeism, and turnover intention.

**Background:**

Abusive supervision has been attributed to suboptimal work performance and reduced productivity among employees in different sectors. While existing nursing literature links abusive supervision to a wide range of work‐related outcomes in the nursing workforce, little is known regarding the mechanism underlying this relationship.

**Design:**

Data for this descriptive study were collected from 770 direct‐care nurses from seven acute care hospitals in the Philippines, utilizing five standardized scales.

**Results:**

Abusive supervision had direct positive effects on absenteeism (*β =* .189, *p* < .001) and intent to leave (*β = *.138, *p* < .001) and a direct negative effect on job engagement (*β = *−.131, *p* < .001). The relationships between abusive supervision and absenteeism (*β = *.175, *p* < .001), intent to leave (*β = *.131, *p *< .001), and work engagement (*β = *−.122, *p* < .001) were partially mediated by stress.

**Conclusions:**

Stress mediated the relationship between abusive supervision and nurses’ work outcomes, including turnover intention, absenteeism, and work disengagement.

**Implications for nursing and health policy:**

The evident connection between abusive supervision, stress, and work‐related outcomes underscores the importance of focusing on enhancing managerial supervisory styles as a potential organizational strategy to enhance staff retention and well‐being.

## BACKGROUND

Nurse turnover, further exacerbated by the COVID‐19 crisis, stands as one of the most critical challenges facing healthcare organizations across many countries worldwide. Global estimates indicate that by 2030, an additional 13 million nurses will be necessary to adequately meet the escalating healthcare demands (International Council of Nurses, 2022). Despite notable growth in the nursing workforce in the United States in recent years, the demand for qualified nurses is projected to approach nearly 1.2 million by 2030 (Hassmiller & Wakefield, 2022). The urgency, cost, and profound impact of nurse turnover on nurses, patients, and healthcare organizations (Bae, 2022; Dall’ Ora et al., 2022) underscore the need to assess various workplace factors that potentially influence turnover. These factors encompass leadership and supervision, job conditions, workload challenges, compensation and benefits, and wellness resources (Cummings et al., 2021; Labrague et al., 2021).

Among these elements, leadership is a powerful determinant of nurse retention. Evidence links effective leadership styles, including authentic, transformational, and ethical leadership, to higher job productivity and increased nurse retention (Al Sabei et al., 2023; Labrague & Obeidat, 2022; Niinihuhta & Häggman‐Laitila, 2022). Despite the relative importance of yielding to effective leadership styles to improve patient and nurse outcomes, reports have demonstrated the increasing presence of the dark side of leadership, including abusive supervision (Bhattacharjee & Sarkar, 2022; Labrague, 2023a; Pradhan & Jena, 2018). Studies on abusive supervision have strongly linked this type to a broad list of job consequences in nurses and organizations (Lavoie‐Tremblay et al., 2016; Mullen et al., 2018).

Abusive supervision is broadly defined as “a subjective evaluation resting on ‘subordinates’ perceptions of the extent to which supervisors engage in the sustained display of hostile verbal and nonverbal behaviors, excluding physical contact” (Tepper, 2000, p. 178). These behaviors may encompass bullying, belittling, demeaning, lying, making negative comments, invading privacy, and isolating employees from others (Fischer et al., 2021; Tepper, 2000). Abusive behaviors are often associated with dispositional or contextual causes. A leader may engage in abusive supervision to conceal their incompetence or if a perceived threat to their position or goal is baffled (Lavoie‐Tremblay et al., 2016; Tepper, 2000). Contextual elements that may drive leaders to exercise abusive supervision include poor organizational culture, the size of the organization, the span of control, the absence of checks and balances, and the lack of policies and guidelines to address abusive behaviors in the workplace (Labrague, 2021).

In nursing, continual exposure to abusive supervision may contribute to a myriad of negative work outcomes, including a decrease in work performance and job engagement, increased turnover intention, and reduced organizational citizenship behavior (Malik et al., 2022; Ozkan, 2022; Whitman et al., 2014). In a systematic review of nursing literature, abusive supervision was associated with various detrimental consequences for nurses, encompassing motivational, relational, cognitive/social‐cognitive, and affective impacts (Labrague, 2023a). Additionally, a leader's abusive behavior may contribute to a work environment where nurses are silenced, and their innovative and creative minds are restricted, resulting in counterproductive work behaviors (Lyu et al., 2019). Some studies attributed such behavior to heightened psychological strain, emotional exhaustion, and poorer health and well‐being (Arefnezhad & Fathi, 2022; Shih et al., 2023). Abusive supervision has also been identified as a strong determinant of poor care quality ratings (Labrague, 2023b; Nia et al., 2022). Mediators such as nurses’ psychological health and well‐being, work attitude, deviant and silent behavior, and work productivity were found to explain how the abusive behavior of a leader influences their subordinate's outcomes (Sharif et al., 2022; Shih et al., 2023). To date, no research has attempted to examine the intermediary effect of stress on the relationship between abusive supervision and direct‐care nurses’ work‐related outcomes (e.g., work engagement, absenteeism, and turnover intention). Understanding this relationship will guide hospital and nursing administrators when designing leadership programs and other institutional initiatives to improve job productivity and enhance nurse retention.

### Theoretical framework

The conservation of resources (COR) theory was used as a theoretical framework in this study. According to the COR theory, individuals strive to acquire, retain, and protect resources that they value, including material goods, personal energy, time, and social support. This theory posits that stress occurs when individuals perceive a threat to their resources or experience actual resource loss (Hobfoll et al., 2000). As a coping mechanism, an individual may confront the situation or withdraw from it to preserve the remaining resources (Holmgreen et al., 2017). In the management context, a leader manifesting an abusive behavior, through their hostile actions or behaviors, may threaten these resources, resulting in higher stress in employees, thus affecting their work and productivity (Hobfoll et al., 2016). Abusive behavior by a leader may threaten employees' psychological well‐being by eroding self‐esteem and confidence, depleting critical social resources through isolation, and consuming energy resources due to heightened stress. This erosion of resources, as per the COR theory, creates a negative spiral effect, impacting individuals' overall resource portfolio and resulting in psychological strain (Holmgreen et al., 2017).

Based on the literature review and the COR theory, these hypotheses were tested: (1) abusive supervision is negatively associated with direct‐care nurses’ job engagement; (2) abusive supervision is positively associated with direct‐care nurses’ absenteeism; (3) abusive supervision is positively associated with direct‐care nurses’ turnover intention; (4) abusive supervision is positively associated with stress; (5) abusive supervision, through the intermediary role of stress, is negatively associated with direct‐care nurses’ job engagement; (6) abusive supervision, through the intermediary role of stress, is positively associated with direct‐care nurses’ absenteeism; and (7) abusive supervision, via the intermediary role of stress, is positively associated with direct‐care nurses’ turnover intention.

## METHODS

### Research aim

The research aimed to test whether stress could mediate the association between abusive supervision and nurses’ work engagement, absenteeism, and turnover intention. The findings of this study have been reported in accordance with the STROBE checklist.

### Design, samples, and setting

This study employed a descriptive design to collect data from direct‐care nurses in seven hospitals in the Philippines, comprising five government‐funded hospitals and two private hospitals. These hospitals were randomly selected from the list of hospitals within the region. Inclusion criteria for nurses were a completed Bachelor of Science in Nursing, current registration/licensure, at least six months of work in their current organization, and consent to participate. Nurses in management positions, including unit supervisors, unit managers, and charge nurses, were excluded. This targeted approach allows for a more nuanced exploration of the effects of abusive supervision on the well‐being and work experiences of frontline nursing staff. The sample size of 765 was determined using an online sample calculator with a 3% margin of error, an alpha value of 0.05, and a 95% confidence level (Soper, 2022). This approach, widely used in numerous studies, proved practical and reliable (Labrague et al., 2020; Sathian et al., 2014). Out of 800 distributed questionnaires, a 96% response rate was achieved, with 770 returned questionnaires.

### Instrumentation

This study used five measures to collect data: the Abusive Supervision Scale (ASS) (Tepper, 2000), the Perceived Stress Scale (PSS), the Utrecht Work Engagement Scale (UWES) (Schaufeli et al., 2002), and two single‐item questions to measure intent to leave and absenteeism. The internal consistency values of the scales were 0.87, 0.80, 0.92, and 0.83.

Nurses’ perceptions of abusive supervision were assessed using the ASS. The scale consisted of 15‐item statements whereby nurses indicated the frequency of abusive behaviors of their nurse managers using a 5‐point Likert frequency scale ranging from 1 (“I cannot remember him/her ever using this behavior with me”) to 5 (“He/she uses this behavior very often with me”). Higher scores in the ASS represent higher abusive supervision. Numerous nursing studies have established the scales’ predictive validity and reliability (Malik et al., 2022; Mullen et al., 2018). Stress was evaluated using the PSS. The 10‐item scale was designed to evaluate the life stress of an individual. Nurses were asked to respond to each item on the scale, ranging from 0 (“never”) to 4 (“often”). The reliability and criterion validity of the scale were previously established in numerous research examining the stress experience of healthcare professionals (Cohen et al., 1988).

Work engagement in nurses was examined using the UWES. The scale consisted of nine items categorized into three domains: vigor, dedication, and absorption. Nurses were required to respond to each item using a 7‐point Likert frequency scale ranging from 1 (“never”) to 7 (“always”). The validity and reliability of the scale were previously examined in numerous studies involving nursing and non‐nursing samples (Osei et al., 2022; Sun et al., 2022).

Nurses’ turnover intentions were examined with a single‐item statement: “I am thinking about leaving this healthcare facility,” using a 5‐point Likert frequency scale ranging from 1 (“strongly disagree”) to 5 (“strongly agree”). This item has been utilized in numerous research involving healthcare and nursing professionals and found to have acceptable reliability and validity indices (Lavoie‐Tremblay *et al.*, 2016; Ozkan, 2022). Absenteeism was examined by asking nurses to quantify the number of absences they had in the past six months. This item has been widely used in many studies across disciplines and found to be reliable and valid (Labrague, 2021).

### Ethical consideration and data collection

The Institutional and Review Board of State University Philippines provided the ethical clearance for the study. The primary researcher sent letters to hospital nurse directors to request approval for data collection. After obtaining permission to conduct the study, the research assistant determined the number of eligible nurses in each hospital based on the formulated eligibility criteria. Before completing the questionnaires, which were enclosed in sealed envelopes, a brief orientation was conducted to explain the purpose and nature of the study, discuss potential risks, and obtain written consent from the participants. To ensure participant anonymity, unique codes were used in the questionnaires instead of personal identifiers. Data were collected from November 2020 to February 2021.

### Data analysis

Missing values were observed in five questionnaires and were subsequently excluded from the data analysis. Descriptive data were quantified using means, frequencies, standard deviations, and percentages. The mediating effects of stress on direct‐care nurses' work outcomes were examined using the three‐stage process by Baron and Kenny (1986). The initial step involved evaluating the direct effect of abusive supervision on direct‐care nurses' turnover intentions, absenteeism, and job engagement. Next, the effect of abusive supervision on the mediator variable, stress, was tested. Finally, the indirect effects of the independent variable on the dependent variable through the mediating variable were assessed using multiple regression analysis.

## RESULTS

Seven hundred seventy nurses returned the questionnaires. Of these, 58.6% (*n* = 451) were female, 56.8% (*n* = 437) were unmarried, 80.1% (*n* = 617) held baccalaureate nursing degrees, and 81.6% (*n* = 628) held tenured positions. More than half the nurses were employed in private (*n* = 404) and tertiary hospitals (*n* = 437), whereas 45.8% (*n* = 353) were employed in medium‐sized healthcare facilities, mostly in urban areas (73.4%, *n* = 565). The nurses' mean ages and years of experience were 28.55 and 5.49 years, respectively.

The mean scale score in the abusive supervision measure was below the median (*m* = 1.563, SD = .689). The mean scale scores for intent to leave, stress, and work engagement were 2.284, 3.091, and 3.332, respectively. The average number of absences incurred for the previous six months was 4.835. Abusive supervision had significant and positive relationships with intent to leave (*r* = .095, *p* < .01), absenteeism (*r* = .195, *p* < .01), and stress (*r* = .078, *p* < .01), and a negative correlation with job engagement (*r* = −.129, *p* < .01). Stress had significant and positive correlations with intent to leave (*r* = .077, *p* < .01) and absenteeism (*r* = .159, *p* < .01), and a significant and negative correlation with job engagement (*r* = −.101, *p* < .01) (Table 1).

**TABLE 1:** Descriptive statistics and the correlations between key study variables.

Variables	Mean	SD	1	2	3	4	5
1. Abusive supervision	1.563	0.689	1				
2. Turnover intention	2.284	1.057	0.095^**^	1			
3. Absenteeism	4.835	5.182	0.195^**^	−0.118^**^	1		
4. Stress	3.091	0.672	0.078^*^	0.077^*^	0.159^**^	1	
5. Work engagement	3.332	0.608	−0.129^**^	−0.001	−0.115^**^	−0.101^**^	1

* *p* < .001,

** *p* < .01.

**FIGURE 1:**
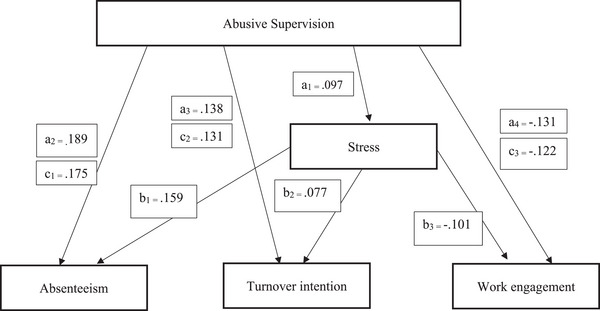
Final model.

Abusive supervision had direct positive effects on absenteeism (*β =* .189, *p* < .001) and intent to leave (*β = *.138, *p* < .001) and a direct negative effect on work engagement (*β = *−.131, *p* < .001). Moreover, abusive supervision had a direct positive effect on stress (*β = *.095, *p* < .01). Stress, the mediating variable, had direct positive effects on intent to leave (*β = *.077, *p* < .05) and absenteeism (*β = *.159, *p* < .001), and a direct negative effect on job engagement (*β = *−.101, *p* < .01). Finally, the mediator variable (stress) partially mediated the relationship between the independent variable (abusive supervision) and the dependent variables [absenteeism (*β = *.175, *p* < .001), intent to leave (*β = *.131, *p* < .001), and work engagement (*β = *−.122, *p* < .001)]. In other words, working with a nurse manager with an abusive supervision style could heighten stress in nurses, which, in turn, increases turnover intention and absenteeism and reduces their job engagement (Table 2; Figure 1).

**TABLE 2:** Effect estimates.

Structural Paths	B	SE	β	*t*	*p*	95% CI LLCI	95% CI ULCI
***Direct effects***							
Abusive supervision							
Absenteeism	1.421	0.266	0.189	5.331	0.001	0.897	1.944
Turnover intention	0.211	0.055	0.138	3.852	0.001	0.104	0.319
Work engagement	−0.115	0.032	−0.131	‐3.659	0.001	−0.177	−0.054
Stress	0.095	0.035	0.097	2.713	0.007	0.026	0.164
Stress							
Turnover intention	0.121	0.057	0.077	2.141	0.033	0.010	0.232
Absenteeism	1.224	0.275	0.159	4.458	0.001	0.685	1.763
Work engagement	−0.091	0.032	−0.101	−2.815	0.005	−0.155	−0.028
***Indirect effects***							
Abusive supervision							
Stress							
Work engagement	−0.108	0.032	−0.122	−3.411	0.001	−0.170	−0.046
Absenteeism	1.317	0.265	0.175	4.966	0.001	0.796	1.837
Turnover intention	0.202	0.055	0.131	3.665	0.001	0.094	0.309

Abbreviations: LLCI, lower limit confidence interval; SE, standard error; ULCI, upper limit confidence interval.

## DISCUSSION

The study's findings supported the hypothesis linking abusive supervision to direct‐care nurses' intent to leave their jobs, absenteeism, and work engagement through the intermediary role of stress. Furthermore, it confirmed the validity of the COR theory. The mean value for the ASS was below the median (m = 1.563), indicating a lower inclination to use abusive supervision as a management style among Filipino nurse managers. When comparing this result to previous studies that utilized Tepper's ASS, we found a lower mean value in the present study compared with those reported in China (Lyu et al., 2019), the United States (Whitman et al., 2014), Malaysia (Low et al., 2021), and Pakistan (Jawad et al., 2021; Malik et al., 2022). One possible explanation for this could be that Filipinos, especially those in higher organizational or societal ranks, place a premium on a virtue called “pakikipagkapwa tao”—concern for others as a means of building good relationships and camaraderie in the workplace, and to achieve social belonging and acceptance (Hawass, 2016). This result aligns with an earlier report in which nurses identified that their nurse managers demonstrated fewer toxic leadership behaviors, a form of abusive behavior, and tended to exhibit more transformational leadership traits (Labrague et al., 2020).

The results of the first, second, and third hypotheses indicate that leaders' abusive supervision has a direct impact on nurses' work outcomes, independent of their demographic characteristics. These findings suggest that when nurses perceive their managers exhibiting abusive supervision, they are more likely to lose interest in their job, have more absences, and desire to leave their organizations. The direct influence of abusive supervision on these critical work outcomes in the nursing workforce underscores the importance of developing positive leadership and management skills among supervisors as a potential strategy to mitigate attrition and enhance nurse retention. Given the ongoing shortage of qualified nurses, it is essential to address all potential factors associated with nurses' disengagement and turnover, including examining the behaviors of nurse managers. This result further aligns with international studies both within (Fisher et al., 2021) and outside the nursing profession (Jawad et al., 2021; Malik et al., 2022), where managers displaying abusive supervision and mismanagement styles, such as hostile verbal and nonverbal reactions, had a significant and adverse impact on their employees' work engagement levels, work productivity, work motivation, and intent to leave their job and the organization.

A key finding of the study was the partial mediating effect of stress on the association between abusive supervision and absenteeism, intent to leave, and job satisfaction, providing partial support for the third, fourth, and fifth hypotheses. This finding suggests that stress can help explain how leaders' abusive behavior may result in adverse work outcomes in direct‐care nurses. This result provides additional support for international studies and holds theoretical significance. During the COVID‐19 pandemic, the abrupt shift to remote work, health concerns, and economic uncertainties likely intensified the impact of abusive supervision on employee stress. Remote work may have blurred the boundaries between personal and professional life, leading to increased demands and challenges in managing work responsibilities (Labrague et al., 2021; Labrague & de Los Santos, 2021). Concerns about job security and health, coupled with the overall uncertainty during the pandemic, may have heightened employees' vulnerability to the negative effects of abusive supervision.

In this context, the mediation role of stress becomes even more significant, as the cumulative stressors related to both abusive supervision and the pandemic could have synergistic effects. The confluence of these stressors may have contributed to higher turnover intention, increased absenteeism, and greater work disengagement among employees (Al Sabei et al., 2022; Labrague et al., 2021). Understanding these dynamics is crucial for organizations seeking to navigate and mitigate the impact of challenging supervisory relationships during times of heightened stress, such as the COVID‐19 pandemic.

This study contributes additional knowledge to the nursing literature regarding the role of stress in explaining the relationship between abusive supervision and outcomes in the nursing workforce. Previous research has identified various potential mediators between abusive supervision and several outcomes, including job burnout, psychological empowerment, employee silence behavior, anxiety and depression, self‐compassion, and self‐efficacy, as well as feedback avoidance, job neglect, turnover intention, work engagement, and organizational citizenship behavior (Arefnezhad & Fathi, 2022; Lyu et al., 2019; Malik et al., 2022; Ozkan, 2022; Whitman et al., 2014). However, none of these studies examined stress as a possible explanation for how this behavior can adversely affect nurse work outcomes. Additionally, a study examined the mediating effect of occupational stress linked abusive supervision to nurses' well‐being rather than absenteeism, turnover intention, or work engagement (Shih et al., 2023).

The mediating effects of stress on the relationship between abusive supervision and a wide range of nursing outcomes provide evidence of the validity of the COR theory. The COR theory posits that stress can emerge when an individual's resources are threatened or lost, or when they are unable to gain resources (Holmgreen et al., 2017). In nursing, these resources may come from various sources, including support from coworkers and management, structural resources for task completion, work stability, a positive relationship with the supervisor, and opportunities for professional growth and advancement (Malik et al., 2022). An abusive behavior of a leader could likely threaten these valuable resources, resulting in stress and affecting work productivity (Fisher et al., 2021; Hobfoll et al., 2016). For example, due to a lack of regard for their subordinates, a leader manifesting abusive supervision may not provide adequate social support and structural resources, leading to frustration and psychological strain. To cope, subordinates might preserve their remaining resources to avoid chronic or prolonged stress by disengaging, increasing absenteeism, and ultimately leaving their jobs (Whitman et al., 2014). This result is crucial, especially considering the significant stress experienced by direct‐care nurses, which was further exacerbated by the coronavirus pandemic. It underscores the need to reevaluate and redesign current stress management initiatives in many healthcare organizations. Given this finding, hospital administrators should prioritize implementing supportive leadership and management training programs, fostering a positive work environment to mitigate stress, enhance job satisfaction, and ultimately reduce turnover and absenteeism rates among staff nurses. Additionally, creating avenues for open communication and psychological support can further contribute to the overall well‐being of nurses and improve retention in the healthcare workforce.

### Study limitations

Caution should be exercised when generalizing and interpreting the results, considering the limitations identified in this study. First, causal inferences may not be possible due to the research design used in this study. Although an appropriate sample size was used to detect statistically significant results based on the conducted sample calculations, the exclusion of nurses from other cities may affect the generalizability of the study results. Second, the use of self‐reporting scales to examine key study variables is another important limitation. While self‐report scales are commonly used in many health and nursing research studies, there is a risk of response bias. Lastly, in future research, other potential factors explaining how abusive supervision influences nurse outcomes should be considered, given the partial mediating effects observed in this study.

### Implications for nursing practice/policy

Given the detrimental outcomes associated with abusive supervision, it is essential to foster positive management styles among nurse managers to enhance nurses' well‐being and ultimately improve retention. This can be achieved through theory‐driven interventions, relevant professional development, human resource strategies, and organizational policies. In two separate systematic reviews (Cummings et al., 2021; Labrague et al., 2021), interventions for leadership development, while shown to improve leadership behaviors and practices in nurse managers, could also potentially reduce toxic behaviors. Other leadership strategies may include leadership mentoring and coaching (Kanninen et al., 2021), leadership simulation (Labrague, 2021), leadership fellowship (MacPhee et al., 2012), and leadership certifications (Foster et al., 2018).

To effectively address abusive supervision in the workplace, Pradhan and Jena (2018) proposed a framework classified into three levels: primary, secondary, and tertiary. Primary interventions are implemented before abusive behavior is reported and include stringent screening of prospective manager candidates for possible aggressive behavior using relevant scales and the implementation of zero‐tolerance policies regarding negative behavior, which should be emphasized during orientation and made visible in work areas. Secondary levels of prevention include activities to be undertaken when abusive supervision is reported, which may include providing relevant training to improve such behaviors (for managers) and counseling for victims of such abuses. Finally, tertiary interventions are implemented as a last resort and may include behavioral change strategies and leadership training.

Addressing stress in nurses, as an important mediator, may result in more desirable outcomes, including higher job engagement and lower turnover intention. Strategies to address stress include the use of mindfulness‐based programs (Green & Kinchen, 2021), cognitive‐behavioral skills training (Terp et al., 2019), relaxation interventions (Veiga et al., 2019), and educational interventions (Xie et al., 2020). The use of technology, such as smartphone‐based stress management programs (Imamura et al., 2021), virtual reality home‐based stress management training (Pallavicini et al., 2022), and simulation‐based interventions (Labrague, 2021), has also been shown to have a positive impact on nurses' stress levels and coping abilities.

## CONCLUSION

This study makes a significant contribution to the nursing literature by highlighting the substantial impact of working under a nurse manager exhibiting abusive supervision on the psychological well‐being of nurses. Consequently, this heightened stress leads to adverse consequences for work‐related outcomes, including decreased job engagement, higher rates of absenteeism, and an increased intention to leave the position. Considering the persisting shortage of qualified nurses, further exacerbated by the ongoing pandemic, it is imperative for nursing administrators to prioritize strategies that enhance retention. This can be achieved by fostering positive leadership behaviors among nurses through theory‐driven approaches, targeted leadership training, and reinforced institutional policies.

## AUTHOR CONTRIBUTIONS

*Study design*: LJL. *Data collection*: LJL. *Data analysis*: LJL. *Study supervision*: LJL. *Manuscript writing*: LJL. *Critical revisions for important intellectual content*: LJL.

## CONFLICT OF INTEREST STATEMENT

The author declares no conflicts of interest.

## FUNDING INFORMATION

This research is not funded.

## ETHICS STATEMENT

Prior to data collection, the research protocol was submitted to the Institutional and Review Board of State University Philippines (109‐2019) for ethical clearance.
